# Integrating Artificial Intelligence into Community Health Nursing Education and Practice: Opportunities, Ethical Challenges, and Future Directions

**DOI:** 10.3390/healthcare14101407

**Published:** 2026-05-20

**Authors:** Bandar Alhumaidi, Talal Ali F. Alharbi

**Affiliations:** 1 Department of Community Health Nursing, College of Nursing, Taibah University, Medina 42353, Saudi Arabia; 2 Department of Community, Psychiatric and Mental Health Nursing, College of Nursing, Qassim University, Buraidah 51452, Saudi Arabia; talal.alharbi@qu.edu.sa; 3 College of Nursing, Sulaiman AlRajhi University, Al Bukayriah 51941, Saudi Arabia

**Keywords:** artificial intelligence, community health nursing, nursing education, AI literacy, ethical challenges, health equity, nursing practice

## Abstract

**Highlights:**

**What are the main findings?**

- AI offers transformative opportunities for community health nursing across predictive analytics, clinical decision support, disease surveillance, and personalized health education.
- Nursing education programs globally lack structured AI curricula, with over 90% of nursing students in the Arab region reporting no formal AI instruction.

**What are the implications of the main findings?**

- Responsible AI integration requires multi-level strategies, including curriculum reform, ethical governance frameworks, and equity-centered design adapted to community health contexts.
- AI should augment rather than replace the relational, culturally sensitive, and holistic care central to community health nursing, preserving the humanistic core of the profession.

**Abstract:**

**Background/Objectives**: Artificial intelligence (AI) is rapidly transforming healthcare. Its integration into community health nursing—a discipline centered on population-level prevention, health promotion, and primary care in community settings—remains insufficiently explored. This narrative review examines the opportunities, ethical challenges, and future directions for integrating AI into community health nursing education and practice. **Methods**: A literature search was conducted across PubMed, CINAHL, Scopus, Web of Science, and IEEE Xplore for publications between January 2017 and March 2026. The initial search yielded 612 records; after the removal of duplicates and screening of titles, abstracts, and full texts against predefined criteria, 58 sources were retained for thematic synthesis, comprising empirical studies, systematic and umbrella reviews, scoping reviews, meta-analyses, and authoritative policy documents. Screening and data extraction were performed by two reviewers, with disagreements resolved by discussion. **Results**: AI offers opportunities for community health nursing across four interconnected domains: clinical decision support for community-based assessments, predictive analytics for population health management, enhanced disease surveillance and outbreak detection, and personalized health education delivery. Significant challenges persist, including algorithmic bias, data privacy concerns, threats to the therapeutic nurse–client relationship, inadequate AI literacy among nursing faculty, and regulatory gaps. Most empirical evidence originates from hospital or general nursing settings; transferability to community contexts is therefore inferred rather than directly demonstrated. **Conclusions**: Responsible integration of AI into community health nursing requires curriculum reform, ethical governance frameworks, faculty development, equitable access, and interdisciplinary collaboration. AI should augment, not replace, the relational and culturally sensitive care that defines this discipline. Given the narrative nature of the review and the limited community-specific evidence, conclusions are framed as a vision of the AI–community health nursing interface rather than a definitive synthesis.

## 1. Introduction

Artificial intelligence, broadly defined as the capacity of computational systems to perform tasks that traditionally require human cognitive processes—including pattern recognition, natural language processing, predictive modeling, and autonomous decision-making—has emerged as one of the most consequential technological forces reshaping contemporary healthcare [1,2]. The convergence of exponentially growing health datasets, advances in machine learning algorithms, and increasing computational power has enabled AI applications that range from diagnostic imaging analysis and drug discovery to clinical decision support systems and population health management platforms [1,3]. Within nursing, the largest healthcare profession globally, AI technologies are being explored across a broad spectrum of practice domains, from acute care monitoring and medication management to patient education and administrative workflow optimization [4,5,6].

Community health nursing occupies a distinctive position within the broader nursing profession. Operating primarily outside hospital walls—in homes, schools, community centers, public health agencies, and occupational settings—community health nurses focus on prevention, health promotion, and the management of health conditions at the population level [7]. Their practice encompasses epidemiological surveillance, health needs assessment, family and home-based care, chronic disease management, immunization campaigns, school health programs, disaster preparedness, and health education targeting vulnerable and underserved populations. The relational, culturally embedded, and often resource-constrained nature of community health nursing practice presents unique opportunities and challenges for AI integration that differ substantially from those encountered in acute care or hospital-based settings.

Despite the burgeoning literature on AI in healthcare and, increasingly, in nursing education and practice, a focused examination of AI’s role specifically within community health nursing remains conspicuously absent. Recent umbrella reviews and systematic reviews have synthesized evidence on AI in nursing broadly [4,5,6], on nursing students’ attitudes and readiness toward AI [8,9,10], and on AI’s applications in clinical nursing care [11,12]. However, the community health nursing context—with its emphasis on social determinants of health, cultural sensitivity, continuity of care across settings, and population-level interventions—demands a tailored analysis that the existing literature has not adequately provided.

This narrative review aims to address that gap by (a) mapping the landscape of AI applications relevant to community health nursing practice; (b) examining the integration of AI concepts into nursing education curricula with particular attention to community health competencies; (c) critically analyzing the ethical challenges that arise when deploying AI in community settings; and (d) proposing evidence-informed recommendations and future directions for research, education, and practice.

## 2. Methods

This narrative review was conducted in accordance with established methodological guidelines for narrative synthesis in health sciences. A comprehensive literature search was performed across the PubMed/MEDLINE, CINAHL, Scopus, Web of Science, and IEEE Xplore databases. The search strategy employed Boolean combinations of the following terms: “artificial intelligence,” “machine learning,” “deep learning,” “natural language processing,” “predictive analytics,” “clinical decision support,” “nursing,” “community health nursing,” “public health nursing,” “nursing education,” “health promotion,” “population health,” “disease surveillance,” “home health,” “ethics,” and “health equity.”

Inclusion criteria encompassed empirical studies, systematic reviews, scoping reviews, umbrella reviews, meta-analyses, narrative reviews, and policy documents published in English between January 2017 and March 2026. Articles were included if they addressed AI applications in nursing practice, nursing education, community health, or public health contexts. Exclusion criteria included conference abstracts without full text, opinion editorials without data, and studies focused exclusively on non-nursing healthcare disciplines without relevance to nursing roles. Reference lists of key articles were hand-searched to identify additional relevant publications. The synthesis was organized thematically around the major domains of AI application in community health nursing and the educational, ethical, and implementation considerations surrounding AI adoption.

**Figure 1:**
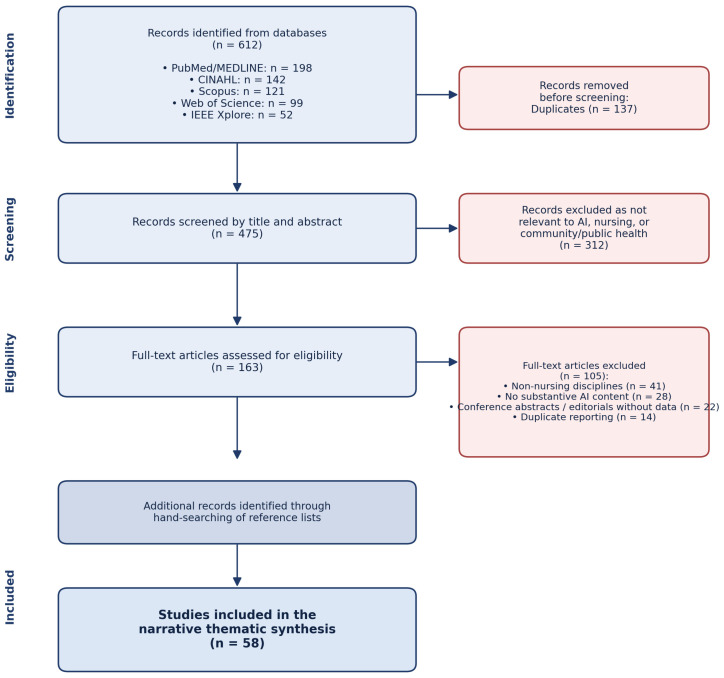
PRISMA-informed flow diagram of the study selection process. The diagram summarizes the identification, screening, eligibility, and inclusion stages of the literature search underpinning this narrative review. Screening and full-text assessment were conducted by two reviewers independently; disagreements were resolved by discussion and, where needed, by consultation with a third reviewer. A formal risk-of-bias appraisal was not performed, consistent with the narrative nature of the review.

A simplified, PRISMA-informed selection process was applied. The initial database search yielded 612 records (PubMed/MEDLINE *n* = 198; CINAHL *n* = 142; Scopus *n* = 121; Web of Science *n* = 99; IEEE Xplore *n* = 52). After the removal of 137 duplicates, 475 records were screened by title and abstract; 312 were excluded as not relevant to AI, nursing, or community/public health. The remaining 163 full-text articles were assessed for eligibility, of which 105 were excluded for the following reasons: focus on non-nursing disciplines (*n* = 41), lack of substantive AI content (*n* = 28), conference abstracts or editorials without data (*n* = 22), and duplicate reporting (*n* = 14). A final pool of 58 sources was retained for thematic synthesis. Hand-searching of reference lists identified additional supporting policy documents and seminal works that were incorporated where relevant. Title/abstract and full-text screening were conducted by two reviewers independently; disagreements were resolved by discussion and, where needed, by consultation with a third reviewer. Of the included sources, the majority addressed AI in nursing or healthcare broadly; only a small subset (approximately one-quarter) reported data from genuinely community-based, primary care, or public health settings, while the remainder informed extrapolations to community contexts. Although a formal risk-of-bias appraisal was not performed—consistent with the narrative nature of the review—the relative weight of higher-quality syntheses (umbrella reviews, systematic reviews with meta-analysis) versus single-site or descriptive studies was considered during synthesis. The full study selection process is also depicted visually as a PRISMA-style flow diagram in Figure 1.

## 3. AI Applications in Community Health Nursing Practice

The potential applications of AI in community health nursing practice span multiple functional domains, each corresponding to core competencies of community health nursing.

It should be acknowledged at the outset that the evidence base supporting these applications is uneven. Of the studies reviewed, only a small proportion were conducted in genuinely community-based, primary care, or public health settings; the majority originated from hospital-based or general nursing contexts. The mapping presented in Table 1 and discussed in the subsections below therefore represents, in part, the authors’ vision of the interface between AI and community health nursing—an interpretive synthesis informed by adjacent evidence rather than a direct empirical demonstration. Where applicable, the text distinguishes findings derived from community contexts from those extrapolated from other nursing or digital health settings. The terms “community health nursing,” “public health nursing,” and “primary care nursing” are used in this review under the broader umbrella of population-focused nursing practice delivered outside acute-care hospital walls; differences in scope across jurisdictions are acknowledged but not exhaustively detailed here.

**Table 1:** AI application domains in community health nursing practice. Representative supporting references by domain: Predictive Analytics [13,14]; Clinical Decision Support [6,12]; Disease Surveillance [15,16,17]; Health Education [18,19,20]; Remote Monitoring [21,22]; Workflow Optimization [5,23]; Mental Health Support [20,24].

Application Domain	Description	Relevance to Community Health Nursing	References
Predictive Analytics	Risk stratification algorithms using demographic, clinical, and social determinants data	Enables proactive outreach; prioritizes resource allocation to high-risk populations	[13,14]
Clinical Decision Support	AI tools assisting in assessment, triage, and care planning during home visits	Reduces cognitive load; enhances diagnostic accuracy in resource-limited settings	[6,12]
Disease Surveillance	ML models analyzing real-time data streams for outbreak detection	Enables rapid response; supports epidemiological investigation	[15,16,17]
Health Education	Adaptive platforms and AI chatbots tailoring content to individual needs	Increases accessibility; addresses health literacy disparities	[18,19,20]
Remote Monitoring	AI-integrated wearables and IoT sensors for continuous vital sign monitoring	Extends nursing reach; supports aging-in-place strategies	[21,22]
Workflow Optimization	NLP documentation systems and automated scheduling algorithms	Reduces administrative burden; increases direct patient care time	[5,23]
Mental Health Support	AI chatbots and sentiment analysis for screening and crisis triage	Reduces stigma barriers; provides 24/7 accessibility	[20,24]

AI-powered predictive analytics represent a transformative tool, enabling the synthesis of vast, heterogeneous datasets—including electronic health records, claims data, social determinants indicators, and environmental monitoring data—to generate risk stratification models that identify individuals and communities at elevated risk for adverse health outcomes [13,14]. For community health nurses, such tools could fundamentally enhance the efficiency and precision of case finding, home visit prioritization, and proactive outreach activities.

AI technologies are increasingly being applied to public health surveillance, offering capabilities that substantially exceed those of traditional reporting-based systems. Machine learning algorithms can analyze diverse real-time data sources—including syndromic surveillance data, social media signals, internet search trends, and environmental monitoring—to detect emerging disease outbreaks faster than conventional methods [15,16]. Natural language processing can extract epidemiologically relevant information from unstructured clinical notes [17], while passively sensed behavioural and physiological signals are increasingly explored for digital phenotyping at the individual and population levels [25].

Community health nurses frequently practice with considerable autonomy, often making complex clinical decisions in resource-limited settings. AI-based clinical decision support systems offer the potential to enhance the quality and safety of these decisions by synthesizing patient data, evidence-based guidelines, and predictive models to generate actionable recommendations [6,12]. AI technologies can also transform health education delivery by enabling adaptive, personalized educational experiences [18,19]. AI-powered chatbots can provide accessible, stigma-free health information for sensitive topics such as mental health, sexual health, and substance use [20]. Comparable applications are being mapped across specialty domains, including cancer nursing [26], and scoping work continues to chart the evolving role of AI across the breadth of nursing practice [27].

## 4. AI Integration in Community Health Nursing Education

The integration of AI into nursing education has emerged as a rapidly growing area of scholarship, with bibliometric analyses revealing an exponential increase in publications since 2020 [28]. Despite widespread recognition of the need to prepare nursing students for an AI-enabled healthcare future, the integration of AI into nursing curricula remains in its early stages globally [10,29], a pattern echoed across scoping and systematic reviews of AI in nursing and health professions education [30,31,32]. A comprehensive systematic review and meta-analysis synthesizing evidence from 111 peer-reviewed articles found that while awareness and positive attitudes toward AI are growing among nursing students and faculty, structured curriculum-based AI education remains notably absent in most programs [10], including limited attention to digital proficiency, health literacy, and the responsible use of generative AI tools [33,34]. In the Arab region specifically, research indicates that over 90% of nursing students report no formal instruction in AI [9].

Evidence from multiple systematic reviews indicates that nursing students generally hold positive attitudes toward AI and express high intentions to adopt AI technology in practice [8,35,36]; comparable findings extend to medical and dental student populations and to the broader nursing science literature [37,38]. However, these positive attitudes coexist with significant knowledge deficits, moderate-to-high AI-related anxiety, and concerns about job displacement, depersonalization of care, and ethical implications [9,35,39,40]. Studies from Saudi Arabia, Palestine, Turkey, Egypt, and Jordan consistently demonstrate a pattern of high awareness but low formal training [9,35,41,42].

Emerging evidence from intervention studies suggests that structured AI educational programs can effectively improve nursing students’ knowledge, skills, and attitudes toward AI [43,44]. A critical bottleneck in AI education for nursing is the readiness of faculty. Research indicates that nursing faculty generally hold neutral-to-positive attitudes toward AI but demonstrate minimal-to-moderate self-rated knowledge and skills [8,10]. Faculty development initiatives are essential for building the instructional capacity needed [44,45].

It is important to acknowledge that most of the literature summarized above addresses AI in general nursing education rather than community health nursing education specifically. The implications drawn for community-focused curricula—including the need for content on social determinants of health, cultural responsiveness, equity-centered AI design, and population-level analytics—therefore reflect the authors’ perspective and analytic synthesis rather than a body of evidence directly generated within community health nursing education. Targeted curricular research situated explicitly within community and public health nursing programs is needed to confirm and refine these recommendations.

## 5. Potential Ethical Challenges and Considerations

The deployment of AI in community health nursing raises a constellation of ethical challenges. Perhaps the most consequential concern is the risk that AI systems will perpetuate or amplify existing health inequities. A seminal study published in Science demonstrated that a widely used commercial algorithm for population health management exhibited significant racial bias, systematically underestimating the health needs of Black patients compared with White patients at equivalent levels of illness severity [46]. For community health nurses, who disproportionately serve marginalized and underserved populations, the deployment of biased algorithms could systematically misdirect resources [47].

The practice of community health nursing is built upon trust. The introduction of AI technologies that collect, analyze, and transmit sensitive personal health data introduces potential threats to this trust [48]. In community settings, where care is delivered in intimate environments involving vulnerable populations, the stakes of data breaches or unauthorized surveillance are exceptionally high. Robust informed consent processes, transparent data governance policies, and strong data encryption protocols are essential prerequisites [49,50,51].

A recurring theme is the concern that technological adoption may erode the humanistic, relational dimensions that constitute the unique contribution of nursing [52,53]. The challenge is to integrate AI in ways that free community health nurses from administrative tasks—thereby increasing time for direct human interaction—while guarding against substituting algorithmic efficiency for nuanced clinical judgment and compassionate presence [3,45,54].

### 5.1. The Central Role of Humans: Why AI Cannot Substitute the Community Health Nurse

A central premise of this review is that AI must be conceptualized as an assistive tool, not as a replacement for the community health nurse. Several intrinsic features of community health nursing render full algorithmic substitution neither feasible nor ethically defensible. First, community health nursing is fundamentally a relational practice grounded in trust, cultural attunement, empathy, and presence—qualities that emerge from embodied human interaction and cannot be simulated by current or foreseeable AI systems [3,52,53]. Second, community health nurses operate as moral agents who exercise contextual judgment in situations characterized by ambiguity, competing values, and social complexity, including domestic violence assessment, end-of-life conversations, immunization hesitancy, and care of stigmatized populations; such judgment requires lived ethical reasoning rather than pattern recognition [52,53]. Third, the nurse’s role as advocate, witness, and bridge between the family, the community, and the formal health system has no algorithmic equivalent: AI can analyze data about a community but cannot stand alongside a vulnerable family in their home. Fourth, accountability in health care—legal, professional, and moral—rests with identifiable human professionals; delegating decision-making to opaque algorithms risks creating accountability vacuums that disproportionately harm marginalized populations [49,55]. For these reasons, the appropriate framing is one of human–AI collaboration in which the nurse remains the decision-maker and the AI functions as a cognitive and operational aide.

### 5.2. Future Scenarios: What Could Happen if AI Controls Community Health Nursing

It is instructive to contrast two contrasting future scenarios. In the first, an “AI-dominant” scenario, decision authority is progressively delegated to algorithmic systems. Risk-stratification engines determine who receives a home visit; chatbots conduct health education and triage; remote sensors and predictive models replace direct nursing assessment for frail older adults; and documentation, prioritization, and care planning are generated automatically. The plausible consequences include de-skilling of the workforce as nurses lose opportunities to develop and exercise clinical judgment; workforce contraction or role downgrading, particularly in resource-limited settings where AI tools are positioned as cheaper substitutes for trained professionals; widening of health inequities when biased algorithms misallocate resources away from already underserved populations [46,47]; erosion of patient trust as care becomes mediated through screens rather than presence; loss of contextual knowledge about families and neighborhoods that no dataset captures; and accountability gaps when adverse outcomes occur [49,55]. Vulnerable groups—older adults, people with low digital literacy, ethnic and linguistic minorities, and rural populations—would bear the greatest burden of such a transition. In the second, “human-centered augmentation” scenario, AI is deployed to handle high-volume, repetitive, or data-intensive tasks (documentation, scheduling, surveillance signal detection, risk flagging, and multilingual education delivery), freeing nurses to spend more time on relational care, complex assessment, advocacy, and community engagement. In this scenario, AI extends nursing reach without diminishing nursing roles, and the workforce evolves toward higher-order competencies in data interpretation, ethical oversight, and community partnership. The actual future will lie somewhere between these poles; which pole it approaches depends on deliberate policy and professional and educational choices made now.

### 5.3. Potential Solutions: Safeguarding the Human Core of Community Health Nursing

Steering toward the human-centered augmentation scenario will require coordinated action across regulatory, professional, educational, organizational, and technological domains. At the regulatory and policy level, jurisdictions should adopt human-in-the-loop and human-on-the-loop requirements for any AI system that influences clinical decisions in community settings, building on emerging instruments such as the EU AI Act and WHO ethics guidance [50,55]. High-risk applications—those affecting eligibility for services, resource allocation, or clinical interventions—should require independent algorithmic auditing for bias and equity impact before and after deployment [46,47]. Workforce-protection clauses should explicitly prohibit the use of AI to justify reductions in licensed nursing positions when patient outcomes have not been demonstrably improved. At the professional level, nursing organizations should issue position statements affirming that final clinical authority and accountability rest with the registered nurse, and they should develop AI competency standards that emphasize critical appraisal of algorithmic output rather than passive compliance [8,10,29]. At the educational level, curricula should cultivate “algorithmic vigilance”: the disposition and skill to question, override, and report AI recommendations when they conflict with clinical judgment or community context [10,45]. Faculty development is a prerequisite, given documented gaps in instructor AI literacy [8,10]. At the organizational level, health services should adopt co-design processes in which community health nurses and community members shape how AI tools are configured, deployed, and evaluated and should establish “AI ethics committees” or equivalent governance bodies for ongoing oversight [13,49,56]. At the technological level, developers should be required to provide explainable outputs, transparent training-data documentation, and accessible mechanisms for clinician feedback and override; AI tools intended for community settings should be validated in those settings rather than only in tertiary hospitals. Finally, at the patient and community level, informed consent processes should disclose when AI is used in decisions affecting care, and patients should have the right to request human-only review. Together, these solutions reframe the question from “can AI replace the nurse?” to “how do we design systems that ensure AI serves the nurse, the patient, and the community?”

## 6. Barriers to Implementation

Beyond ethical considerations, several practical barriers impede the integration of AI into community health nursing. Infrastructure limitations, including inadequate internet connectivity in rural settings, insufficient hardware, and limited access to electronic health record systems, represent fundamental prerequisites that remain unmet in many community health contexts [9,14]. Institutional resistance, stemming from organizational inertia and the absence of institutional AI policies, further constrains adoption [8,10]. The shortage of faculty with sufficient AI expertise represents a critical human capital barrier [10,57]. Regulatory and legal frameworks governing AI in healthcare decision-making remain underdeveloped [51,55]. Financial barriers also merit attention, as community health organizations often operate under tight budgetary constraints.

A further barrier, and one closely linked to the substitution debate, is workforce anxiety. Documented concerns among nursing students and practicing nurses about job displacement, role downgrading, and de-skilling [9,35,39,40] act as a brake on adoption when they are not openly addressed. If AI is introduced without transparent commitments that it will augment rather than replace nursing roles, frontline engagement is likely to be defensive rather than collaborative, undermining the co-design and evaluation processes on which safe deployment depends. Conversely, where institutions communicate clearly that AI will absorb administrative load and free nursing time for direct community engagement, acceptance and constructive critique are more likely. Addressing this human-capital barrier, therefore, requires not only technical capacity building but also explicit workforce-protection messaging, role-evolution pathways, and meaningful inclusion of nurses in governance.

## 7. Toward a Framework for Responsible AI Integration

Drawing upon the evidence synthesized in this review, a multi-level framework for the responsible integration of AI into community health nursing can be proposed. At the educational level, nursing programs should introduce AI literacy as a core competency, beginning with foundational concepts in pre-licensure education and progressing to advanced applications in graduate programs [10,29,58]. At the practice level, the deployment of AI tools should be guided by principles of equity-centered design, ensuring that AI solutions are developed with and for the communities they serve [46,50]. Community health nurses should be positioned as active participants in the co-design, testing, and evaluation of AI tools. At the governance level, ethical frameworks specifically adapted to community health contexts are needed, addressing algorithmic transparency, bias auditing, data sovereignty, informed consent, and accountability [49,50,55].

A fourth and explicitly human-centered layer should be added to this framework. Across all three preceding levels, integration must be governed by the principle that AI augments rather than replaces the community health nurse. Operationally, this principle translates into (i) a default human-in-the-loop requirement for any AI output that informs assessment, triage, prioritization, or care planning, with the nurse retaining final authority and clear override rights; (ii) workforce-protection commitments from employers and policymakers that link AI deployment to role enrichment—for example, redeploying time saved on documentation toward home visits, community engagement, and complex case management—rather than to staff reductions; (iii) algorithmic vigilance training so that nurses are equipped to recognize bias, drift, and inappropriate generalization in AI tools deployed in their communities [10,45,46]; (iv) transparent communication with patients and communities about when and how AI contributes to decisions affecting them, alongside the right to request human-only review; and (v) a continuous monitoring loop in which nurse-reported and patient-reported experiences with AI tools feed back into governance and procurement decisions. This human-centered layer is what distinguishes responsible integration from technological displacement and operationalizes the “AI as assistant, not substitute” principle in concrete institutional practice.

## 8. Future Directions

Several priority areas for future research emerge from this review. First, implementation science studies are needed to evaluate the real-world effectiveness, feasibility, and sustainability of AI tools in community health nursing settings. Second, longitudinal research is required to assess the impact of AI-enhanced curricula on graduates’ competencies, practice behaviors, and patient outcomes. Third, participatory research approaches involving community members in AI tool co-design should be prioritized [13,56]. Fourth, investigations into AI’s influence on the nurse–client relationship are warranted. Fifth, comparative effectiveness research examining different AI implementation models can guide resource allocation. Finally, policy research examining regulatory, legal, and workforce implications is essential [7,55].

A further priority is the empirical study of the human–AI workforce interface itself. This includes prospective evaluation of the two scenarios outlined in Section 5.2, with explicit measurement of workforce composition, scope-of-practice changes, de-skilling indicators, role enrichment, and patient-reported relational quality before and after AI deployment. Studies should also test the effectiveness of specific safeguards—such as mandatory human-in-the-loop policies, workforce-protection clauses, algorithmic vigilance training, and patient rights to human-only review—in preserving the relational core of community health nursing. Comparative case studies across health systems with differing regulatory regimes (for example, the EU AI Act framework versus less prescriptive jurisdictions) would help identify which governance configurations best protect against the AI-dominant scenario while still capturing efficiency gains. Finally, qualitative research with community members, particularly from marginalized groups, is needed to understand patient preferences regarding AI involvement in their care and the conditions under which AI-mediated services either erode or enhance their trust in community health nursing.

## 9. Limitations

Several limitations should be acknowledged. First, this is a narrative review and not a systematic review; although a transparent, PRISMA-informed search and screening process was followed, the synthesis is interpretive rather than exhaustive. Second, the search was restricted to five databases and to English-language publications, which may have led to the omission of relevant studies indexed elsewhere or published in other languages. Third, no formal quality or risk-of-bias appraisal of the included studies was conducted, in keeping with the narrative format. Fourth, and most importantly, the evidence specifically generated within community, public, or primary care nursing settings is limited; many of the conclusions and framework components are informed by extrapolation from hospital-based, general nursing, or broader digital health literature and should therefore be read as a vision of the AI–community health nursing interface rather than as a definitive evidence map. Fifth, given the rapid pace of AI development, some technologies and applications discussed here may evolve substantially in the near future. Finally, this review reflects the perspectives and analytic choices of the authors and may not capture all relevant viewpoints, particularly those of community members and frontline community health nurses, whose voices remain underrepresented in the current literature.

## 10. Conclusions

Artificial intelligence holds significant promise for enhancing the efficiency, precision, and reach of community health nursing practice, from predictive population health analytics and real-time disease surveillance to personalized health education and AI-augmented clinical decision-making. However, realizing this promise requires a deliberate, equity-centered, and ethically grounded approach that acknowledges the unique characteristics of community health nursing practice—its focus on vulnerable populations, its reliance on trust and therapeutic relationships, its operation in resource-constrained settings, and its commitment to social justice and health equity. AI should be conceptualized not as a replacement for human nursing expertise but as a powerful augmentation tool that, when responsibly designed, implemented, and governed, can amplify the capacity of community health nurses to fulfill their vital public health mission. Given the narrative nature of this review and the limited body of empirical evidence generated specifically within community health nursing, these conclusions should be interpreted with appropriate caution. They are best understood as a working vision of the AI–community health nursing interface, intended to stimulate further targeted empirical inquiry rather than to prescribe definitive practice or curricular standards. The central message is that the future of community health nursing in the age of AI will be determined less by what technology can do and more by the deliberate choices of educators, regulators, employers, professional organizations, and nurses themselves to keep human judgment, presence, and accountability at the heart of community-based care.

## Data Availability

No new data were created or analyzed in this study. Data sharing is not applicable to this article.

## Abbreviations

AI	Artificial Intelligence
CDSS	Clinical Decision Support System
NLP	Natural Language Processing
IoT	Internet of Things
